# Fahr's Syndrome With Neurocognitive Dysfunction Due to Hypoparathyroidism: A Case Report

**DOI:** 10.1155/crie/6355371

**Published:** 2025-07-15

**Authors:** Faezeh Sehatpour, Shahrzad Mohseni, Mahnaz Pejman Sani

**Affiliations:** Endocrinology and Metabolism Research Center, Endocrinology and Metabolism Clinical Sciences Institute, Tehran University of Medical Sciences, Tehran, Iran

**Keywords:** basal ganglia calcification, case report, dementia, Fahr's syndrome, hypoparathyroidism

## Abstract

**Background:** Fahr's syndrome is a rare neurodegenerative condition characterized by bilateral progressive calcification of the basal ganglia and other brain structures. Due to overlapping symptoms, it can be misdiagnosed as other neurological disorders.

**Case presentation:** A 68-year-old man was presented to the emergency department with an exacerbating decline in the level of consciousness and dysarthria over a 20-day period. On admission, the laboratory examinations revealed a low level of calcium and parathyroid hormone. Brain imaging findings showed bilateral calcifications in the basal ganglia, pulvinar region of the thalami, and dentate nuclei. In addition, a prolonged QTc interval on his electrocardiogram (ECG) indicated hypocalcemia. After receiving calcium gluconate 10%, the calcium level and QTc interval stabilized, and the patient's level of consciousness gradually improved.

**Conclusion:** Fahr's syndrome due to hypoparathyroidism should be suspected in any patient with neurological symptoms and hypocalcemia. Hence, early identification and management of hypoparathyroidism can prevent progression of calcification and improve patients' quality of life and prognosis.

## 1. Introduction

Fahr's syndrome is a rare neurodegenerative disorder with a prevalence of <1/1,000,000 [1]. It was first described by a German neurologist, Fahr, in 1930 by detecting an elderly person with dementia, motor disorders, and extensive calcification of the basal ganglia, and other brain structures [2]. It is characterized by bilateral progressive calcium deposits in various brain regions, including the basal ganglia, thalamus, hypothalamus, hippocampus, cerebral cortex, subcortical white matter, striatum, pallidum, and dentate nuclei [1, 3]. These intracranial calcifications can disrupt emotional, extrapyramidal, and cerebellar brain functions, leading to various neuropsychological manifestations [1]. Research indicates that the severity of psychiatric disorders, but not neurological ones, is associated with the extent of calcification [1, 4, 5].

Fahr's syndrome results from underlying conditions such as vascular, neoplastic, infectious, or inflammatory factors [6]. One of the main causes of Fahr's syndrome is hypoparathyroidism, which can lead to calcification of the basal ganglia in an estimated period of 8–10 years [7]. Hence, early detection and management of hypoparathyroidism can lead to prevent calcification and neurological disorders [8]. In contrast, Fahr's disease is a rare, genetically dominant inherited disorder, though autosomal recessive inheritance and sporadic cases have been reported. The SCL20A2, PDGFB, PDGFRB, and XPR1 genes are most commonly affected. It often appears during the fourth or fifth decade of life, although it can appear during childhood [1, 7, 9].

In the present study, we report a case of Fahr's syndrome secondary to hypoparathyroidism, which presented with neurocognitive disorders.

## 2. Case Presentation

A 68-year-old male patient was admitted to the emergency department due to an exacerbated decline in the level of consciousness and dysarthria over 20 days. On admission, he was disoriented, aphasic, and a fracture of the left humerus following minor trauma. He had a history of cerebrovascular accident (CVA) in December 2023 that resulted in cognitive dysfunction and dementia. Moreover, he had a history of type 2 diabetes, ischemic heart disease, and hip fracture. Upon admission, he was receiving empagliflozin/metformin at 5/500 mg twice a day, furosemide at 40 mg twice a day, apixaban at 5 mg twice a day, clopidogrel at 75 mg daily, memantine at 10 mg daily, carvedilol at 6.25 mg daily, and spironolactone at 25 mg daily.

Physical examination revealed a blood pressure of 130/85 mmHg, a heart rate of 84 beats per minute, a body temperature of 36.9°C, and an oxygen saturation level of 95% in room air.

Initially, his Glasgow Coma Scale (GCS) score was assessed as 9 out of 15. During the neurological evaluation, the patient was disoriented and didn't respond to sound but was responsive to painful stimulation. Both pupils were mid-sized and reactive to light. Plantar reflexes were downward. Both Trousseau's sign and Chvostek's sign were positive. Motor strength was 4/5 on the right and unevaluable on the left side due to fracture. Physical exam for meningeal irritation was also negative. Additionally, the power of the lower extremity motor neurons could not be evaluated because of his current condition.

**Figure 1:**
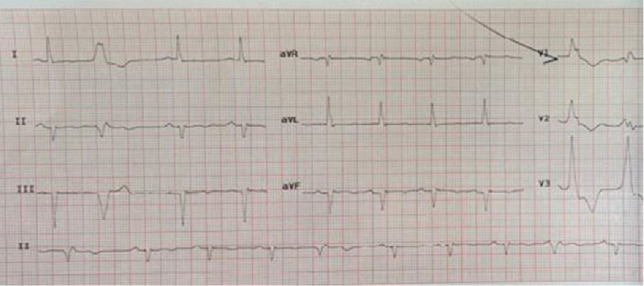
Electrocardiogram shows a sinus rhythm, left axis deviation, multiple premature ventricular complex (PVC), Q wave in inferior lead, T wave flattening, and QTc prolongation.

**Figure 2:**
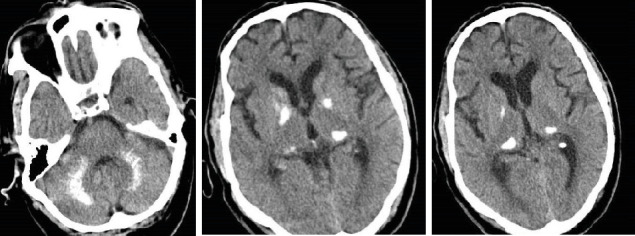
Noncontrast axial computed tomography image shows bilateral calcification of basal ganglia, pulvinar region of thalami, and dentate nuclei.

**Figure 3:**
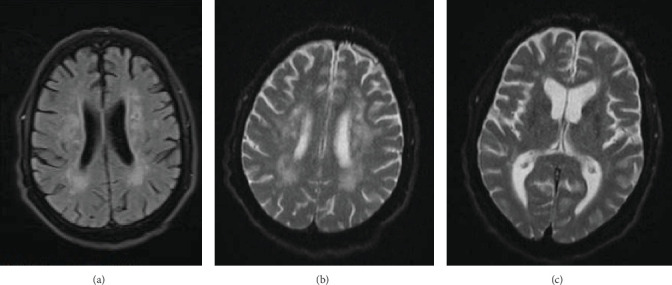
Magnetic resonance imaging (MRI): bilateral calcification of basal ganglia, pulvinar region of thalami, and dentate nuclei (A, B). Bilateral old lacunar infarct in centrum semiovale and bilateral abnormal signal intensity foci in the periventricular white matter and centrum semiovale (C).

Initially, laboratory investigations showed hypocalcemia, hypoparathyroidism, hypomagnesemia, low level of vitamin D, and hyperphosphatemia (Table 1). Electrocardiogram (ECG) demonstrated QTc prolongation (622 ms) suggestive of hypocalcemia (Figure 1). His brain noncontrast computed tomography (CT) scan revealed bilateral calcification in the basal ganglia, pulvinar region of the thalami, and dentate nuclei (Figure 2). Moreover, magnetic resonance imaging (MRI) of the brain revealed an old lacunar infarct in the bilateral centrum semiovale, as well as bilateral abnormal signal intensity foci in the periventricular white matter and centrum semiovale, in favor of old small vessel changes, rather than calcifications as reported in the CT scan (Figure 3). The electroencephalogram (EEG) examination showed a mild diffuse slow pattern, suggesting a nonspecific diffuse cerebral dysfunction.

Two vials of 10% calcium gluconate in 50 mL of 5% dextrose were infused over about 10 min as a bolus dose. Then, 11 vials of 10% calcium gluconate in 1000 mL of 5% dextrose water were administered with the infusion rate of 100 mL/h. Afterwards, the infusion rate was adjusted based on the clinical and biochemical responses. For the treatment of hypomagnesemia, a bolus dose of 2 g (16 mEq) of magnesium sulfate was infused over 2 h, followed by a maintenance infusion of 4 g (32 mEq) over 12 h, which was continued until serum magnesium concentrations stabilized. As a result, both the serum calcium level and the QTc interval on ECG were normalized (QTc: 410 ms). Once the patient's level of consciousness improved, oral calcium carbonate at 500 mg every 4 h and oral calcitriol at 0.25 mcg every 6 h were administered. Finally, after 10 days of hospitalization, the patient was discharged in stable condition with the prescription of oral calcium carbonate and calcitriol therapy.

## 3. Discussion

We report a case of an individual with hypoparathyroidism recently diagnosed with neurocognitive impairment and calcifications of the basal ganglia and dentate nuclei suggestive of Fahr's syndrome.

Fahr's syndrome is a rare neurodegenerative disorder that is associated with a variety of etiological manifestations such as endocrine disorders, infectious diseases, and neurological conditions [1].

One of the most common endocrine etiologies of Fahr's syndrome is hypoparathyroidism. In this condition, an abnormality in calcium/phosphate homeostasis leads to abnormal calcium deposition in the basal ganglia, thalamus, dentate nucleus, cerebellum, subcortical white matter, cerebral cortex, and hippocampus of the brain [1, 3]. However, physiological calcifications in the basal ganglia show a prevalence of only 1.3%, but the presence of calcifications should raise suspicion of a pathological condition such as Fahr's syndrome [10].

Disruption in the corticostriatal tract due to nerve calcification negatively affected the transmission of sensory input from the cerebral cortex to the central components of the basal ganglia [11]. The involved brain regions are responsible for a variety of neuropsychiatric features, including schizophrenia like psychosis, impairment in memory and concentration, dementia, extrapyramidal symptoms, cerebellar dysfunction, and speech problems [3].

Hyperphosphatemia and hypocalcemia secondary to hypoparathyroidism (whether primary, secondary, or pseudohypoparathyroidism) can precipitate aberrant calcification. Hypocalcemia may lead to various clinical manifestations,including paresthesia, spasm, cardiovascular irregularity, positive Chvostek sign, seizures, parkinsonism, extrapyramidal manifestations, cataracts, as well as QTc interval prolongation and cardiac arrhythmias [4, 5, 7, 12]. Hence, immediate management of hypocalcemia is essential. For this purpose, 20 mL of 10% calcium gluconate diluted in 100 mL of 5% dextrose should be infused over about 10 min as a bolus dose. Following the bolus dose to maintain plasma calcium levels, 50 mL (5 vials) of 10% calcium gluconate in 500 mL of 5% dextrose should be infused over 12–24 h, depending upon the clinical and biochemical responses. It is important to avoid direct intravenous infusion, as this increases the risk of phlebitis or tissue damage if extravasation occurs [13]. Vitamin D is also a vital component in calcium metabolism, and plays a significant role in the occurrence of Fahr's syndrome. Therefore, treatment of low levels of vitamin D is essential [1, 7].

Prior to the admission, our patient had been taking furosemide. Furosemide functions by inhibiting the sodium–potassium–chloride cotransporter (NKCC2) in the thick ascending limb of the loop of Henle, thereby decreasing sodium reabsorption. Since the NKCC2 also plays a role in calcium reabsorption, its inhibition by furosemide results in reduced calcium reabsorption and increased calcium excretion in the urine [14, 15]. Generally, this calcium loss would trigger an increase in PTH levels. However, in our case, despite furosemide use, PTH levels remained low while phosphorus levels were elevated, indicating the presence of an underlying pathological condition.

Our patient had a history of CVA, and evidence of lacunar infarction was detected in the brain MRI. This may potentially be attributable to extensive calcium and mineral deposits within affected vessels, which could lead to decreased vascular tone and hemodynamic changes caused by calcification in the media tonica. Calcification in arteries, arterioles, and capillaries, especially in smaller vessels, may obstruct the lumen and contribute to ischemia [16, 17]. In addition, the accumulation of activated astrocytes and microglia around calcified deposits indicates a mild inflammatory process [3]. Further research is necessary to clarify the exact relationship between Fahr's syndrome and cerebrovascular events.

## 4. Conclusion

Prompt diagnosis and management of hypocalcemia, especially in severe cases, can reduce the risk of developing abnormal calcification. Treatment with oral calcium and the active form of vitamin D is essential in cases of primary hypoparathyroidism with severe hypocalcemia or in symptomatic patients, especially those with neurological or cardiovascular symptoms. Intravenous bolus injection of calcium gluconate followed by continuous infusion should be considered as an emergency management in critical cases.

## Figures and Tables

**Table 1:** Laboratory examinations.

Test name	Value in the patient	Unit	Reference range
WBC	8.6	×1000/mm^2^	4.5–12.5
Hemoglobin	11.6	g/dL	14–18
MCV	76.4	fL	
PLT	341	×1000/mm^2^	150–400
BUN	26	mg/dL	8–20
Cr	1.36	mg/dL	0.7–1.4
Random BS	137	mg/dL	70–100
Sodium	144	meq/L	135–145
Potassium	3.8	meq/L	3.6–5.2
Calcium	5.4	mg/dL	8.5–10.5
Magnesium	1.6	mg/dL	1.8–2.6
Phosphorus	9.9	mg/dL	2.6–4.5
AST	26	U/L	<40
ALT	15	U/L	<37
ALP	321	U/L	80–300
Albumin	4.7	g/L	3.5–5
Bilirubin (total)	1.5	mg/dL	Up to 1.1
Bilirubin (direct)	0.3	mg/dL	Up to 0.3
PTH	19.5	pg/mL	11–81
OH-vitamin D 25	16.3	ng/mL	20–50 (optimal level)
Ferritin (ng/mL)	234	ng/mL	21.8–274
Vitamin B12	191.2	pg/mL	187–883
Folic acid	8.5	ng/mL	5.6–45.8
Iron	34	Micg/dL	40–168
TIBC	291	Micg/dL	250–450

Abbreviations: ALP, alkaline phosphatase; ALT, alanine aminotransferase; AST, aspartate aminotransferase; BS, blood sugar; BUN, blood urea nitrogen; Cr, creatinine; MCV, mean corpuscular volume; PLT, platelet; PTH, parathyroid hormone; TIBC, total iron-binding capacity; WBC, white blood cell.

## Data Availability

All the data have been presented within the manuscript.
